# *Smilax china* L. Extract Alleviates Metabolic-Associated Fatty Liver Disease by Regulating Gut Microbiota and Bile Acid Metabolism

**DOI:** 10.3390/metabo16010031

**Published:** 2025-12-26

**Authors:** Shiyuan Cheng, Huijun Li, Zhiying Sun, Yue Xiong, Jing Li, Jiaying Tian, Yue Shen, Li Shen, Jingyu Yang, Yuying Yang, Dan Liu, Qiong Wei, Chao Huang, Xiaochuan Ye

**Affiliations:** 1 Hubei Key Laboratory of Resources and Chemistry of Chinese Medicine, School of Pharmacy, Hubei University of Chinese Medicine, Wuhan 430065, Chinachuan9069@hbucm.edu.cn (X.Y.); 2 Hubei Shizhen Laboratory, Hubei University of Chinese Medicine, Wuhan 430065, China

**Keywords:** *Smilax china* L., metabolic-associated fatty liver disease, gut microbiota, metabolomics, bile acid metabolism

## Abstract

**Background:** Metabolic-associated fatty liver disease (MAFLD) is prevalent in individuals with liver disease; however, it lacks effective therapeutic approaches. *Smilax china* L., a traditional Chinese medicinal herb, possesses excellent anti-inflammatory and antioxidant activity. This research aimed to explore the therapeutic effects of *Smilax china* L. extract (SCE) on MAFLD and to elucidate the pharmacological mechanisms. **Methods:** A rat model of MAFLD was induced through a high-fat diet (HFD), and the model rats subsequently received SCE as a therapeutic intervention for six weeks. The analysis involved 16S rDNA sequencing, untargeted fecal metabolomics, and targeted bile acid metabolomics to investigate the effects of SCE on the gut microbiota and bile acid metabolism. **Results:** Hepatic steatosis and lipid accumulation were significantly alleviated by the SCE treatment. SCE treatment modulated the gut microbiota disorder, by enhancing the relative abundance of the beneficial gut microbiota, including *Clostridium*, *Oscillospira*, and *Romboutsia*. Untargeted fecal metabolomics revealed a significant enrichment of the metabolites in secondary bile acid biosynthesis. Targeted bile acid metabolomics revealed that SCE reversed the abnormal fecal bile acid metabolic profile, such as HDCA, LCA, and T-β-MCA. These changes activated FXR and PPARα receptors to improve the lipid metabolism by regulating bile acid synthesis. **Conclusions**: Our study provides evidence that SCE alleviates MAFLD through regulation of the gut microbiota, bile acid metabolism, and activation of the FXR/PPARα pathway, illustrating the mechanism of action of SCE in MAFLD from a novel perspective, and further highlights its therapeutic potential.

## 1. Introduction

MAFLD is a widespread liver condition that affects approximately a quarter of the global population [1]. As a leading cause of chronic liver disease worldwide, the pathological hallmark of MAFLD is the accumulation of fat in the liver. This condition may progress to steatohepatitis, cirrhosis, and hepatocellular carcinoma [2,3]. The incidence of MAFLD is increasing globally, placing a significant socio-economic burden on the healthcare economy [4]. The rising prevalence of MAFLD is driven primarily by chronic nutrient excess, which directly promotes hepatic steatosis and instigates systemic metabolic dysfunction [5]. MAFLD patients exhibit metabolic dysfunction, which commonly co-occurs with T2DM and CVD, synergistically exacerbating adverse clinical risks [6]. The pathogenic mechanisms underlying MAFLD are multifactorial and remain incompletely characterized [7].

In recent years, studies have shown that gut bacteria are closely related to the occurrence and development of MAFLD [8,9]. In patients with MAFLD, dysbiosis of intestinal flora disrupts the intestinal barrier function and increases the intestinal permeability, which leads to the release of bacterial products such as LPS and pro-inflammatory cytokines into the liver, triggers and maintains inflammatory responses, affects host lipid metabolism, and further participates in the pathologic process of MAFLD [10,11].

There is growing evidence that improving bile acid metabolism can treat MAFLD [12]. BA changes may result in a reduction in hepatic Farnesoid X receptor (FXR) activity, which increases the intracellular sludge of bile acids [13]. Hepatic BA production proceeds through two principal biosynthetic routes, where CYP7A1 is the rate-limiting enzyme in the classical pathway that catalyzes the conversion of cholesterol to bile acids [14]. The classical bile acid synthesis pathway begins with the CYP7A1-mediated 7α-hydroxylation of cholesterol, followed by CYP8B1-mediated side-chain oxidation, accounting for nearly 75% of the total BA production. In contrast, the alternative pathway begins with CYP27A1-catalyzed 27-hydroxylation of cholesterol, succeeded by CYP7B1-mediated hydroxylation, playing a vital part in retaining metabolic homeostasis [15]. Abnormalities in bile acid synthesis and metabolism lead to metabolic diseases such as obesity and chronic liver disease [16].

Studies demonstrate that the gut microbiota affects peroxisome proliferator-activated receptor alpha (PPARα) activity [17,18]. Research has shown that the concurrent stimulation of both PPARα and FXR improves liver sensitivity to insulin while correcting the lipid metabolic disturbances characteristic of NASH [19]. In addition, the interaction between PPARα and FXR may have a synergistic effect and improve MAFLD [20]. Thus, regulation of the gut microbiota–FXR/PPARα may offer a novel treatment strategy for MAFLD.

As an important Chinese herbal medicine and food in Chongqing, *Smilax china* L. (Liliaceae) possesses multifunctional biological activities, particularly anti-inflammatory, antioxidant, and hypoglycemic effects [21,22,23]. The chemical components of *Smilax china* L. mainly include flavonoids, saponins, tannins, and polysaccharides. Previous research reports have shown that *Smilax china* L. polysaccharides can improve MAFLD [24]. However, whether *Smilax china* L. ethanolic extract could have a therapeutic effect on HFD-induced MAFLD rats and its underlying mechanism remains unexplained.

The focus of this research was to investigate the effects and to explore the potential mechanisms of *Smilax china* L. ethanol extract (SCE) on MAFLD induced by HFD in rats. The mechanism exploration was carried out by combining 16S rDNA sequencing and metabolomics techniques. Furthermore, we quantitatively assessed hepatic expression levels of FXR, PPARα, and critical bile acid metabolism-associated targets in liver tissues. Our findings the first experimental verification that SCE alleviates MAFLD through regulation of gut microbial and bile acid metabolic pathways.

## 2. Materials and Methods

### 2.1. Materials, Chemicals, and Reagents

Serum levels of LDL-C, HDL-C, AST, and ALT were detected using commercial ELISA kits from Shanghai Fusheng Industrial Co., Ltd. (Shanghai, China), and LPS was purchased from Bioswamp Industrial Co., Ltd. (Wuhan, China). The kits for TNF-α, IL-6, and IL-18 were purchased from Elabscience Industrial Co., Ltd. (Wuhan, China). Biochemical kits for TC, TG, GSH-Px, SOD, and MDA were obtained from Elascience Industrial Co., Ltd. (Wuhan, China). Chromatographic-grade formic acid acetonitrile, and methanol came from Sigma-Aldrich (Darmstadt, Germany).

Primary antibodies of FXR (Cat. SC-25309, 1:1000), CYP7A1 (Cat. SC-518007, 1:1000) were obtained from Santa Cruz Biotechnology Co., Ltd. (Dallas, CA, USA), and an antibody of Lamin B1 (Cat. AF5161, 1:1000) was obtained from Affinity (Affinity Biosciences, USA). The CYP7B1 (Cat. 24889-1-AP), PPARα (Cat. 66826-1-Ig), and CYP27A1 (Cat. 14739-1-AP) were obtained from Proteintech Industrial Co., Ltd. (Chicago, IL, USA). The CYP8B1 (A25847) was obtained from ABclonal Biotechnology Co., Ltd (Wuhan, China).

### 2.2. Extraction Procedure of SCE

The *Smilax china* L. samples were collected from Tongcheng County, Xian’ning City, Hubei Province, China. The plant material was authenticated as *Smilax china* L. rhizome by Professor Xiaochuan Ye at Hubei University of Chinese Medicine in Wuhan, China. In this procedure, 15 kg of dried *Smilax china* L. was soaked in 150 L of 60% EtOH (1:8) for 1 h and then refluxed for 2 h twice. All extracts were pooled and subsequently concentrated to a final volume of 3.8 L, and liquid extract was stored at −20 °C until use.

### 2.3. Chemical Analysis of Smilax china L. Extract

UPLC-Q-TOF-MS (Waters, Milford, MA, United States) was used to analyze the phytochemical composition of SCE. Separation was achieved using an ACQUITY UPLC HSS T3 column, with the following settings: mobile phase flow rate (0.4 mL/min), column temperature (30 °C), and autosampler temperature (5 °C). The binary mobile phase system comprised water (A) and acetonitrile (B), with gradient elution programmed as: 0–4 min (88→85% A), 4–15 min (85→30% A), 15–16 min (30→88% A), and 16–20 min (88% A) a mobile phase velocity (0.45 mL/min) [25,26].

### 2.4. Animal Experiment

Male Sprague Dawley (SD) rats weighing 180–200 g were sourced from Hubei Provincial CDC (Wuhan, China) and housed in SPF circumstances at the Experimental Animal Center of Hubei University of Chinese Medicine. Animals were kept within controlled conditions (12 h light/dark cycle, 24 ± 2 °C, 50 ± 5% humidity). All experimental procedures were approved by the Ethics Committee of Hubei University of Chinese Medicine (approval number: HUCMS00303455, registered on 1 March 2023).

After a one-week adaptation phase, six rats were randomly selected as the control group, while the others received a high-fat diet (composition: 10% lard, 5% egg yolk powder, 5% sucrose, % cholesterol, and 0.5% sodium cholate) for 6 weeks to establish the MAFLD rat model. The MAFLD model rats were divided into 5 groups (*n* = 6 per group): the HFD (water), Atorvastatin (10 mg/kg, Pfizer Pharma Ltd., USA), low-dose SCE (SCE-L), middle-dose SCE (SCE-M), and high-dose SCE (SCE-H). Based on the 2020 *Chinese Pharmacopoeia* and some studies, rats in the SCE-L, SCE-M, and SCE-H groups received gavage administration of 8.1, 16.2, and 32.4 g/kg of SCE (equivalent to the herbal dose), respectively. During the 6-week treatment phase, a normal diet was provided to the control rats, while the five other groups were given high-fat feed.

Post-study, sterile fecal specimens were stored at −80 °C. The rats were anesthetized with pentobarbital sodium, and arterial blood was collected and centrifuged (3000 rpm, 15 min, 4 °C) for serum isolation. The serum aliquots were stored at −80 °C for long-term preservation. The excised livers were precisely weighed for the calculation of the hepatic-to-body weight ratio, with selected tissue portions reserved for pathological assessment.

### 2.5. Histopathology

The partial left hepatic lobule samples underwent fixation in 4% paraformaldehyde at 72 h, and then the trimmed tissues were transferred to a 15% sucrose solution for dehydration. Next, paraffin embedding was performed, and 8 μm slices were prepared. Hematoxylin–eosin (HE) and Oil Red O staining were applied to liver tissue. In addition, the HE samples were analyzed to determine the histopathological diagnosis score by using the MAFLD activity score (MAS). Steatosis, inflammation, and ballooning were scored to determine the MAS, following previous studies [27,28].

### 2.6. Biochemical Analysis

Biochemical assay kits were tested for the analysis of TG as well as TC in the liver and serum samples. Biochemical assay kits were also used for the determination of GSH-Px, SOD, and MDA. The rat ELISA kit was used to detect the levels of IL-18, LDL-c, HDL-c, ALT, IL-6, TNF-α, LPS, and AST in the serum. The experiment was performed according to the kit instructions.

### 2.7. 16S rDNA Sequencing and Analysis

The control, HFD, and SCE-H were chosen for later gut microbiota examination based on their MAFLD treatment effectiveness. We isolated fecal DNA using the E.Z.N.A.^®^ DNA Detection Kit (Omega Bio-tek, Norcross, GA, USA). For microbial community analysis, the microbial 16S rRNA genes were amplified using universal primers. The resulting PCR amplicons were then purified with AMPure^®^ PB magnetic beads and precisely quantified using the Qubit 4.0 Fluorometric system (Thermo Fisher Scientific Inc., Waltham, MA, USA). The obtained experimental data were subsequently analyzed through bioinformatics processing on the Majorbio Cloud Platform. Using the OTU/ASV data, we generated rarefaction curves and computed alpha diversity metrics (Shannon and Simpson indices) using Mothur software (V1.30.1) [29]. The microbial community similarity across samples was evaluated through principal coordinate analysis (PCoA) utilizing vegan (v2.5-3). Treatment-related variations were quantified using PERMANOVA within the same vegan package, while microbial markers were distinguished using the linear discriminant analysis effect size (LEfSe) [30]. The present analysis was conducted to detect the bacterial taxa (from phylum to genus level) showing marked abundance contrasts between groups, applying thresholds of LDA score > 4 and *p* < 0.05.

### 2.8. Metabolomics Analysis

#### 2.8.1. Feces Samples Preparation

We added 50 mg of a fecal specimen to a 2 mL centrifuge tube with 400 μL of ice-cold solvent (methanol/water, 4:1 *v*/*v*) and 0.02 mg/mL L-2-chlorophenylalanine (internal standard). Cryomilling (60 Hz, 6 min) followed by sonication-assisted extraction (50 kHz, 5 °C, 20 min) were performed at −10 °C using a Wonbio-96c homogenizer (Wonbio Biotechnology Co., Ltd., Shanghai, China). Following 30 min incubation at −20 °C, the samples were centrifuged (13,000× *g*, 4 °C, 15 min), and the clarified supernatant was subjected to analysis.

#### 2.8.2. LC-MS/MS Determination

Metabolite profiling was achieved through LC-MS/MS detection implemented on the Thermo UHPLC-Q Exactive HF-X mass spectrometer (Thermo Fisher Scientific Inc., Waltham, MA, USA) separated by an ACQUITY HSS T3 chromatographic column. For chromatographic separation, the following mobile phases were utilized: eluent A, water/acetonitrile (95:5, *v*/*v*), and eluent B, acetonitrile/isopropanol/water (47.5:47.5:5, *v*/*v*/*v*). Here, 0.1% (*v*/*v*) formic acid was added to each eluent, with separation at 0.40 mL/min and a 40 °C column temperature.

#### 2.8.3. Data Processing and Analysis

The acquired UHPLC-Q datasets were analyzed using Progenesis QI software (V2.0) (Waters, Milford, MA, USA). Subsequent multivariate statistical analyses were performed using R (v1.6.2 “ropls” package), including PCA and OPLS-DA, followed by a 7-round cross validation to assess the model robustness. Significantly altered metabolites were determined according to VIP > 1 and *p* < 0.05. To identify relevant pathways, KEGG pathway enrichment analysis of differentially expressed metabolites was used (http://www.genome.jp/kegg/ (accessed on 8 January 2025)).

### 2.9. Quantification of Fecal BAs

The 47 individual BA standards were accurately weighed, dissolved in methanol, and serially diluted to prepare working solutions at varying concentrations. The BA information is shown in Table S1. We weighed 25 mg of a fecal sample, adding 20 μL of working solution 1 (2000 ng/mL) and 380 μL of the extraction solution (methanol/water = 4:1). The sample was homogenized using a cryomill (6 min, −10 °C, 50 Hz), sonicated (30 min, 5 °C, 40 kHz), and incubated at −20 °C for 30 min. After centrifugation (15 min, 4 °C, 13,000 rcf), a 200 μL aliquot of the supernatant was obtained for analysis.

The analysis was performed on an ExionLC AD system with a Waters BEH C18 column (150 × 2.1 mm, 1.7 μm) maintained at 50 °C. A sample volume of 5 μL was injected. The mobile phase system comprised 2 eluents: (A) water and (B) acetonitrile, each containing 0.1% formic acid. Sample analysis was performed on an AB SCIEX QTRAP 6500+ (Foster City, CA), with detection in the negative ion mode. The key parameters were set as follows: curtain gas (CUR), 35; collision gas (CAD), medium; ion spray voltage (IS), −4500 V; temperature (TEM), 550 °C; ion source gas 1 (GS1), 50; and ion source gas 2 (GS2), 50.

### 2.10. Quantitative Real-Time PCR (q-RT PCR) Analysis

Hepatic RNA was separated using a Spin Column Animal Total RNA Purification Kit (Songon Biotech, Shanghai, China), followed by first-strand cDNA generation with the RevertAid Kit (Thermo Fisher Scientific Inc., Waltham, MA, USA). Target genes (*CYP7A1*, *CYP7B1*, *CYP27A1*, *CYP8B1*, *PPARα*) were amplified in triplicate using a StepOnePlus Real-Time PCR System. The relative mRNA expression was determined using the 2^^(–ΔΔCT)^ method and normalized to the endogenous control GAPDH. The primer sequences are provided in Table S2.

### 2.11. Western Blotting

A 50 mg fecal sample was treated with 1000 μL of PMSF, containing protease and phosphatase inhibitors (MedChemExpress, Shanghai, China). Following centrifugation at 10,000× *g* (4 °C, 12 min), protein quantification was performed via BCA (Elabscience, Wuhan, China). Nuclear and cytoplasmic proteins were extracted utilizing a separation kit (NE-PER, Thermo Fisher Scientific Inc., Waltham, MA, USA, 78833) for subsequent FXR detection. After electrophoresis of 24 mg samples using 10% polyacrylamide gels (EpiZyme, Shanghai, China), proteins were transferred to PVDF membranes and incubated with skimmed milk powder under ambient temperature for 50 min. Antibodies against GAPDH, FXR, Lamin B1, CYP7A1, CYP7B1, CYP27A1, CYP8B1, and PPARα were utilized to hybridize the membranes overnight at 4 °C. Following incubation with a secondary antibody for 1 h, the protein bands were detected using an ECL reagent (Biopmk, Wuhan, China) and quantified through densitometry using both AlphaEase FC (Alpha Innotech, California, USA) and ImageJ software (V1.53).

### 2.12. Statistical Analysis

All statistical analyses were performed using GraphPad Prism 9.0 (GraphPad Software, La Jolla, CA, USA). Multigroup comparisons were examined using analysis of variance. The microbial diversity was assessed in terms of the alpha diversity (Majorbio platform) and beta diversity (Bray–Curtis dissimilarity) [29]. Bacterial genus-level differences were identified using Kruskal–Wallis tests, while Spearman’s correlation analysis was applied for association studies. Statistical significance was defined as *p* < 0.05.

## 3. Results

### 3.1. Chemical Constituents of Smilax china L. Extract

**Figure 1:**
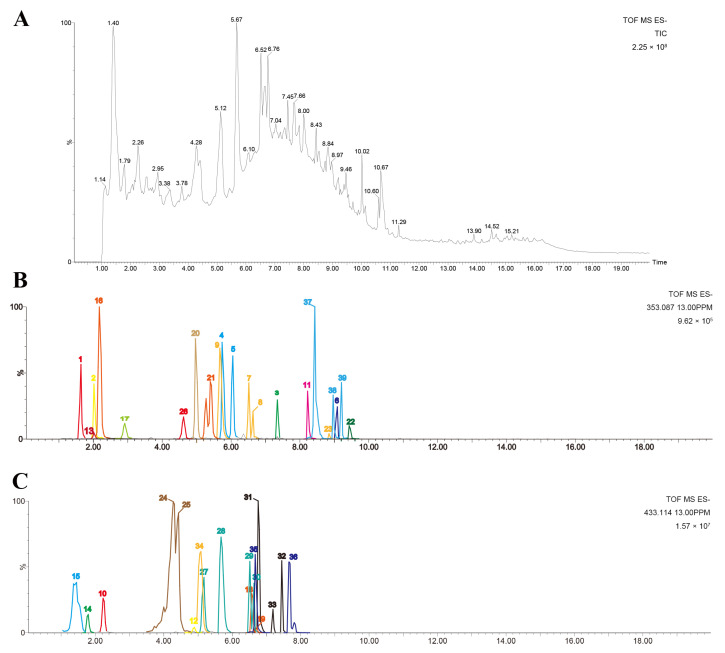
Chemical profiles of SCE analyzed using UPLC-Q-TOF-MS. (**A**) Total ion chromatogram of SCE; (**B**,**C**) extracted ion chromatograms of identified compounds in negative mode.

The chemical components in SCE were analyzed via UPLC-Q-TOF-MS. Through a comprehensive analysis combining fragmentation patterns, standard comparisons, and the literature data, we identified 35 compounds in positive ionization mode (Figure S1 and Table S3) and 39 compounds in negative ionization mode (Figure 1 and Table S4). There were 22 flavonoid components, six steroid saponins, six polyphenols, and five phenylpropanoids in the negative ion mode, which included components from all positive ion modes.

### 3.2. SCE Alleviates Liver Lipid Accumulation and Liver Injury in MAFLD Rats

**Figure 2:**
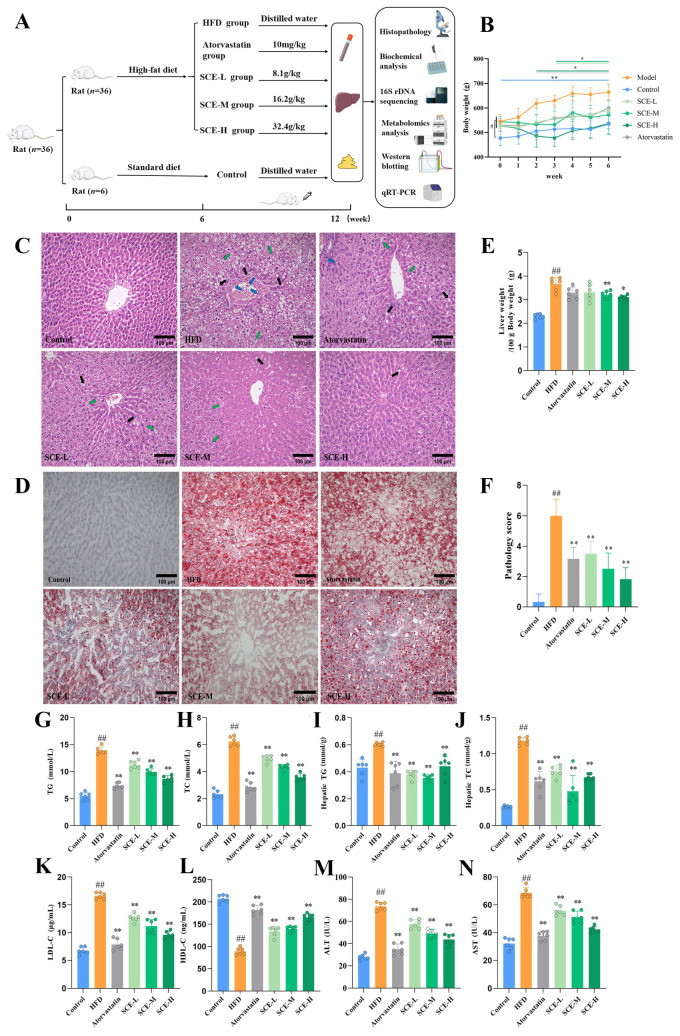
SCE alleviated hepatic steatosis and live injury in MAFLD rats. (**A**) Animal experimental design. (**B**) Rat body weight (^#^ *p* < 0.05, compared with the control group, at week 0, * *p* < 0.05, and ** *p* < 0.01, compared with the HFD group at each week). (**C**) HE staining in liver (×200, black arrows represent hydropic degeneration; green arrows represent lipid vacuoles; blue arrows represent inflammation). (**D**) Oil red O staining in liver (×200). (**E**) Hepatic index. (**F**) Pathology score. (**G**,**H**) Serum levels of TG (**G**) and TC (**H**). (**I**,**J**) The levels of TG (**I**) and TC (**J**) in rat liver tissues. (**K**–**N**) Serum levels of LDL-C (**K**), HDL-C (**L**), ALT (**M**), and AST (**N**) (*n* = 6). Data represent mean ± SD. ^#^ *p* < 0.05, and ^##^ *p* < 0.01, compared with the control group. * *p* < 0.05, and ** *p* < 0.01, compared with the HFD group.

Figure 2B demonstrates that the body weight at the end of modeling (0 week) was significantly higher (*p* < 0.05) in each group than in the control group, and the prolonged intake of SCE led to a significant weight reduction compared with the HFD group (*p* < 0.05). The SCE-H and atorvastatin exhibited significant weight reduction by week 2, whereas the weight-lowering effects in the SCE-L and SCE-M groups became apparent from week 3 (*p* < 0.05). Figure 2C illustrates the liver histological modifications in the rats. The liver lobules of the control group were well structured, the hepatic cords were neatly arranged, and there was no hepatocellular ballooning. In contrast, the HFD resulted in disintegration of the HFD group’s hepatic cord and lobule morphology, with steatosis of more than 1/2 of the hepatocytes per unit area. Compared with the HFD group, the SCE groups showed reduced hepatic lipid accumulation and partial restoration of liver lobule architecture, demonstrating SCE’s ameliorative effects on MAFLD pathology. Oil Red O staining revealed that SCE therapy markedly attenuated the over-accumulation of lipids in hepatocytes (Figure 2D). In contrast to the HFD group, the liver weight in SCE-M and SCE-H rats was much lower (*p* < 0.05, Figure 2E). The pathology score revealed notably elevated values in the HFD group compared with all other experimental groups (*p* < 0.01, Figure 2F). Serum TC, TG, LDL-C, AST, ALT, liver TG, and TC contents of the HFD rats were obviously increased relative to the control, and these indicators were reduced in all the treated groups (*p* < 0.01, Figure 2G–K,M,N). The serum HDL-C level of the HFD group decreased relative to the control, and the HDL-C levels were significantly higher after SCE treatment (*p* < 0.01, Figure 2L). These findings present compelling evidence for the therapeutic potential of SCE in improving both liver steatosis and liver function in MAFLD.

### 3.3. SCE Improves Oxidative Stress and Ameliorates Inflammation in MAFLD Rats

**Figure 3:**
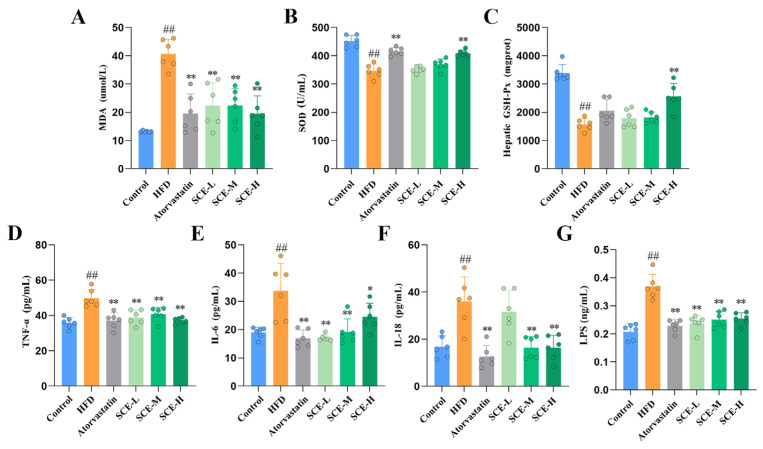
SCE improved inflammation and oxidative stress in MAFLD rats. (**A**,**B**) Serum levels of MDA (**A**) and SOD (**B**). (**C**) Liver levels of GSH-Px. (**D**–**G**) Serum levels of TNF-α (**D**), IL-6 (**E**), IL-18 (**F**), and LPS (**G**). Data represent mean ± SD. ^##^ *p* < 0.01, compared with the control group. * *p* < 0.05, and ** *p* < 0.01, compared with the HFD group.

The biomarkers of oxidative stress were measured to assess the effects of the HFD and SCE on systemic oxidative stress. Relative to the control group, the HFD group exhibited significantly depressed serum SOD and liver GSH-Px, with increased serum MDA levels; SCE treatment remarkably elevated the serum SOD and liver GSH-Px content, in contrast to the decreased serum MDA content of MAFLD rats (*p* < 0.01, Figure 3A–C). Relative to the control group, the serum levels of TNF-α, IL-6, IL-18, and LPS were remarkably elevated in HFD rats. Treatment with SCE markedly decreased these indices in MAFLD rats (*p* < 0.05, Figure 3D–G). In conclusion, these results validate that SCE can ameliorate oxidative stress and inflammation in rat MAFLD.

### 3.4. SCE Affects the Composition of the Gut Microbiota of MAFLD Rats

**Figure 4:**
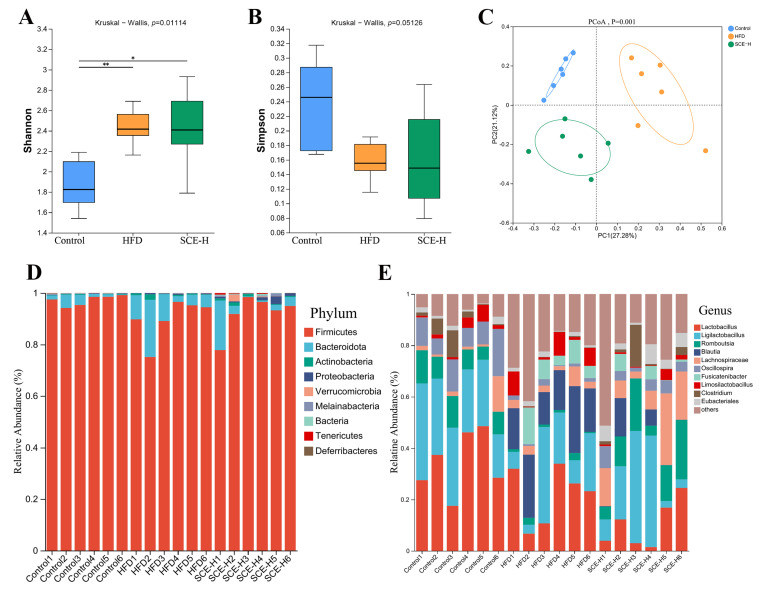
SCE regulated the diversity and composition of rat intestinal microbiota. (**A**) Shannon index. (**B**) Simpson index. (**C**) Principal coordinate analysis (PCoA) plot. (**D**) Relative abundance of intestinal microbiota at the phylum level from the three groups. (**E**) Relative abundance of intestinal microbiota at the genus level. Data are presented as the mean ± SD (*n* = 6). * *p* < 0.05, and ** *p* < 0.01, compared with the HFD group.

To study the influence of SCE on the gut microbiota, we conducted 16S rDNA sequencing on fecal samples to assess the microbial composition and abundance. As depicted in Figure 4A, the Shannon index was obviously higher in both the HFD and SCE-H group relative to the control (*p* < 0.05). In contrast, the Simpson index remained largely unchanged across groups (Figure 4B), indicating that the high-fat diet influenced alpha diversity. Principal coordinate analysis (PCoA) showed that SCE treatment influenced gut microbiota beta-diversity, with the control and SCE-H groups forming tighter clusters compared with the control and HFD groups (Figure 4C). This suggests that SCE induced microbial composition shifts similar to those in the control. There was no visible change at the phylum level between the control, HFD, and SCE-H (Figure 4D). The main changes occurring at the genus level are shown in Figure 4E. These results demonstrate that SCE modulates the gut microbiota primarily at the genus level, without significantly altering its overall structure, including alpha diversity and phylum-level abundance.

**Figure 5:**
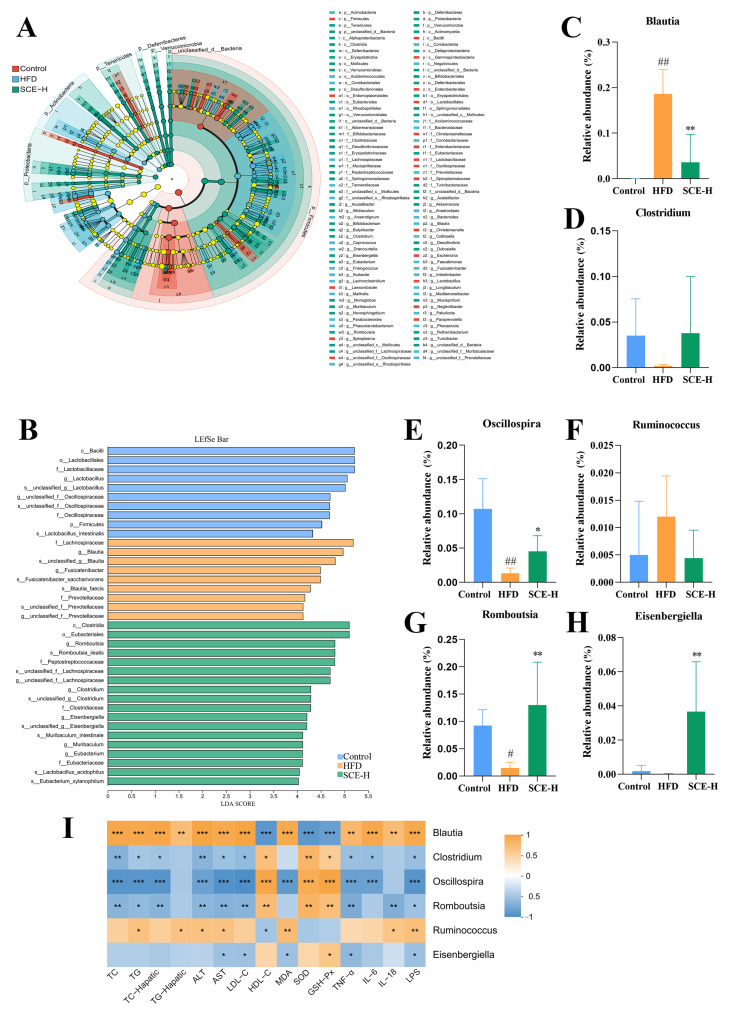
Linear discriminant analysis effect size (LEfSe) analysis of differential gut microbiota. (**A**) LEfSe taxonomic cladogram. (**B**) Histogram of the LEfSe scores and relative abundance of the significantly altered bacteria from the three groups. (**C**–**H**) Gut microbiota species relation abundance. (**I**) Heatmap of Spearman’s correlation between bacteria and biochemical indicators at six genus levels. A positive correlation is displayed as orange, while a negative correlation is shown in blue. Data are presented as the mean ± SD (*n* = 6). ^#^ *p* < 0.05, and ^##^ *p* < 0.01, compared with the control group. * *p* < 0.05, ** *p* < 0.01, and *** *p* < 0.001, compared with the HFD group (In I, compared between bacteria and biochemical indicators).

With a linear discriminant analysis (LDA) score > 4 as the screening standard, we applied LEfSe methods to identify biomarkers in both bacterial community groups. As observed from Figure 5A,B, at the genus level, in comparison with the control group, Blautia was significantly increased, while Oscillospira and Romboutsia were noticeably decreased in the HFD group (*p* < 0.05). However, they were markedly recalled through SCE treatment (*p* < 0.05, Figure 5C,E,G). Moreover, SCE therapy also significantly increased Eisenbergia (*p* < 0.05, Figure 5H) and improved the reduction in Clostridium and the enrichment of Rumiinococcus in HFD rats (Figure 5D,F).

Furthermore, the results of the functional relationship between the altered bacteria and MAFLD-related biochemical indicators using Spearman’s correlation analysis showed that six altered bacteria (Blautia, Clostridium, Oscillospira, Romboutsia, Ruminococcus, and Eisenbergiella) were significantly correlated with the biochemical indicators (*p* < 0.05, Figure 5I). For example, Clostridium, Romboutsia, and Oscillospira were positively correlated with SOD, GSH-Px, and HDL-C and negatively associated with TG, TC, ALT, AST, LPS, MDA, TNA-α, IL6, and LDL-C. Meanwhile, Blautia and Ruminococcus were negatively correlated with SOD, GSH-Px, and HDL-C and positively correlated with TG, TC, ALT, AST, LPS, MDA, TNF-α, IL-6, IL-18, and LDL-C, suggesting that they may be MAFLD-specific bacteria.

### 3.5. SCE Affects Metabolites in MAFLD Rats

**Figure 6:**
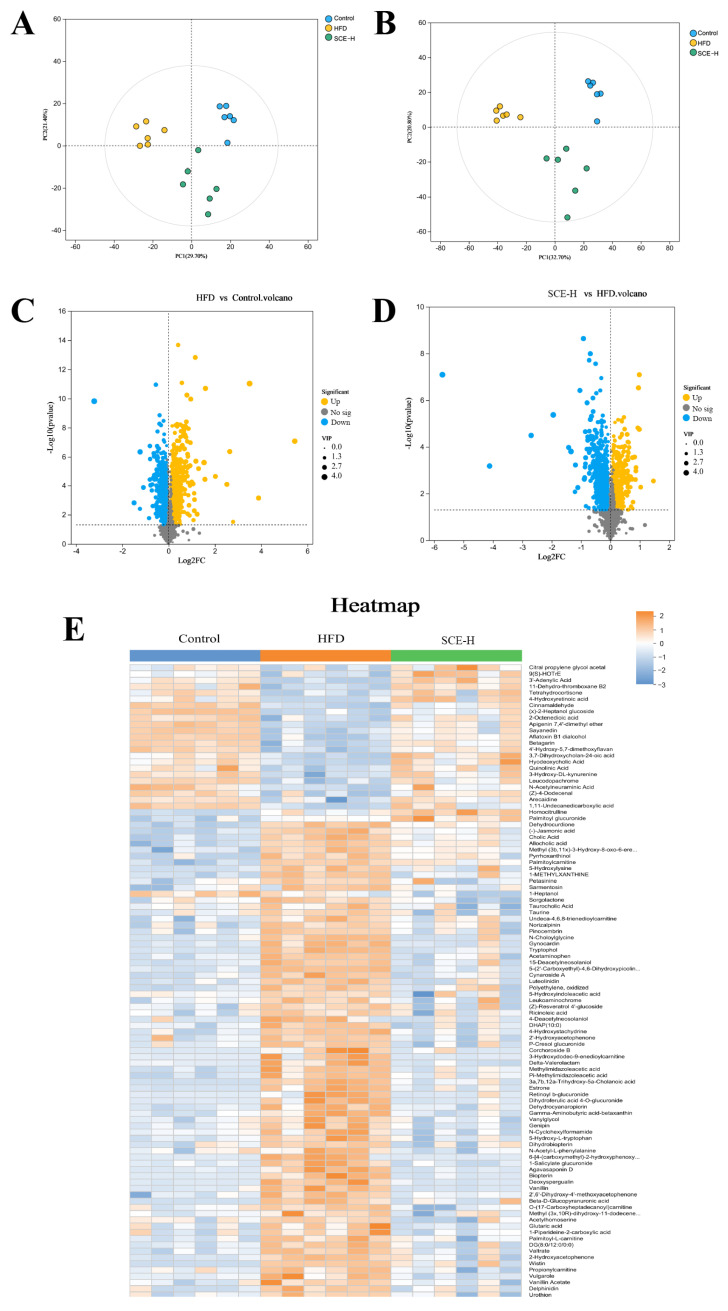
Multivariate statistical analysis of feces metabolomics in the control, HFD, and SCE groups (negative ion mode and positive ion mode, *n* = 6). (**A**,**B**) PCA score plot. (**C**,**D**) Volcanic plot of differentially expressed metabolites of HFD vs. control groups (**C**) and SCE-H vs. HFD groups (**D**). (**E**) Heat map of identified differentially expressed metabolites between the control, HFD, and SCE-H groups.

To evaluate the influence of SCE on rat endogenous metabolites, we performed nontargeted metabolomics profiling of fecal samples from the control, HFD, and SCE-H groups. The PCA results demonstrated good discrimination between the metabolic profiles of the control, HFD, and SCE-H groups (Figure 6A,B).

As shown in the volcano plot (Figure 6C,D), the comprehensive metabolite profile is presented in negative and positive ionization modes, with orange and blue spots indicating substantially increased and decreased metabolites, correspondingly. The size of each dot represents its VIP score. The selection of significantly altered metabolites between the control, HFD, and SCE groups was based on the concurrent application of two statistical thresholds: a *p* < 0.05 from Student’s *t*-test and a VIP > 1 from the OPLS-DA model. Based on MS/MS fragment data, we obtained 99 known differentially expressed metabolites in the HFD versus the control and SCE-H (Figure 6E and Table S5).

**Figure 7:**
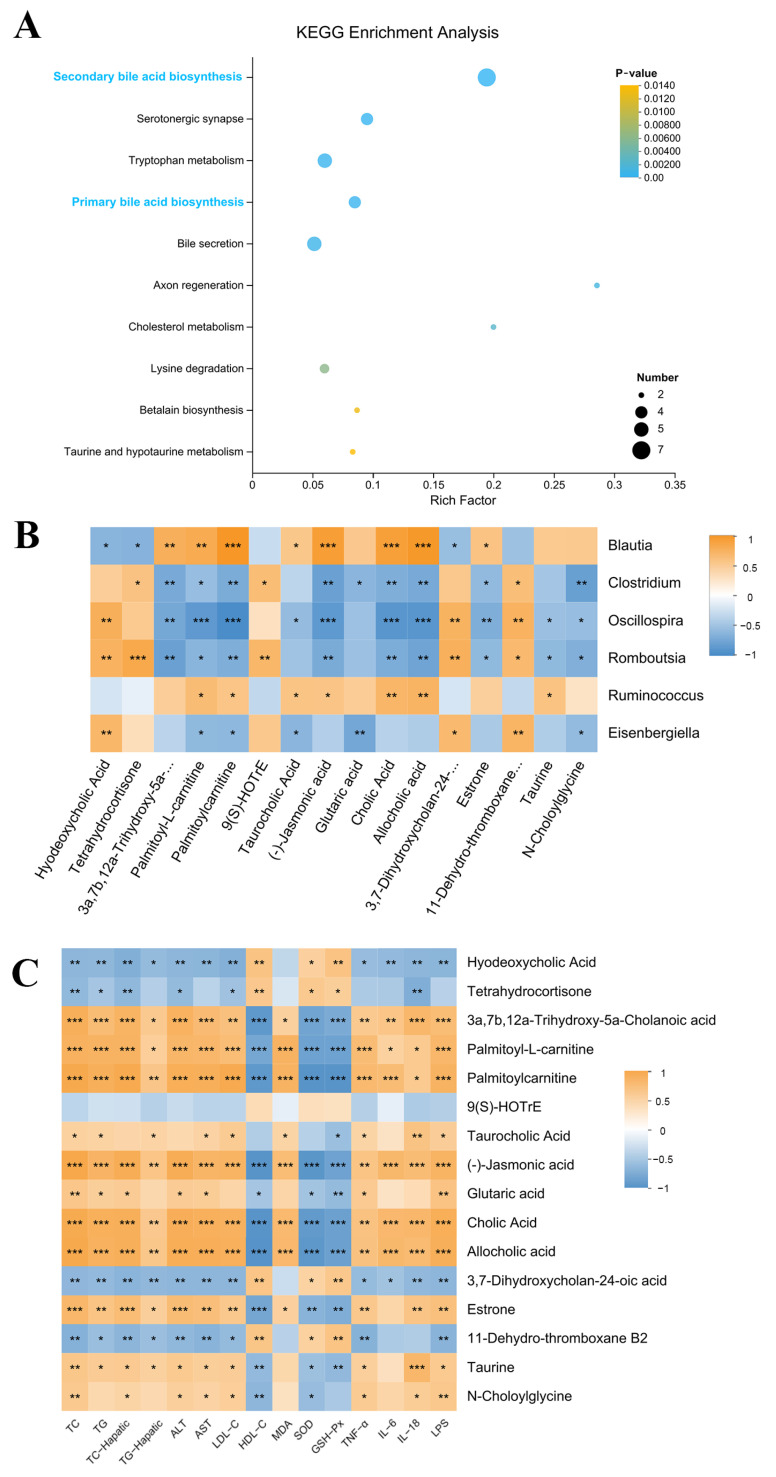
KEGG pathway enrichment analysis and differentially expressed metabolite correlation analysis (**A**) KEGG pathway enrichment analysis. (**B**,**C**) Spearman’s correlation analysis. A positive correlation is displayed as orange, while a negative correlation is shown in blue. The significance is indicated as * *p* < 0.05, ** *p* < 0.01, and *** *p* < 0.001.

Furthermore, potential metabolic pathways associated with SCE-H intervention were investigated using KEGG pathway enrichment analysis. These metabolites were abundant mainly in secondary bile acid biosynthesis (Figure 7A).

We screened 16 metabolites related to lipid metabolism from 99 differentially expressed metabolites via the KEGG pathway. Hyodeoxycholic acid, tetrahydrocortisone, 3a,7b,12a-Trihydroxy-5a-Cholanoic acid, 9(S)-HOTrE, 3,7-Dihydroxycholan-24-oic acid, and 11-Dehydro-thromboxane B2 metabolites all markedly declined in the HFD group and dramatically increased after SCE-H treatment. In contrast, cholic acid, palmitoyl-L-carnitine, palmitoylcarnitine, taurocholic acid (TCA), (-)-jasmonic acid, glutaric acid, allocholic acid, estrone, taurine, and N-Choloylglycine were enhanced in the HFD group and decreased after SCE-H treatment (Table S6).

In addition, Spearman’s correlation analyses were conducted to detect connections between differentially expressed gut microbiota and metabolites (Figure 7B) and between differentially expressed metabolites and biochemical indicators (Figure 7C).

### 3.6. SCE Effects on Bile Acid Metabolism in MAFLD Rats

**Figure 8:**
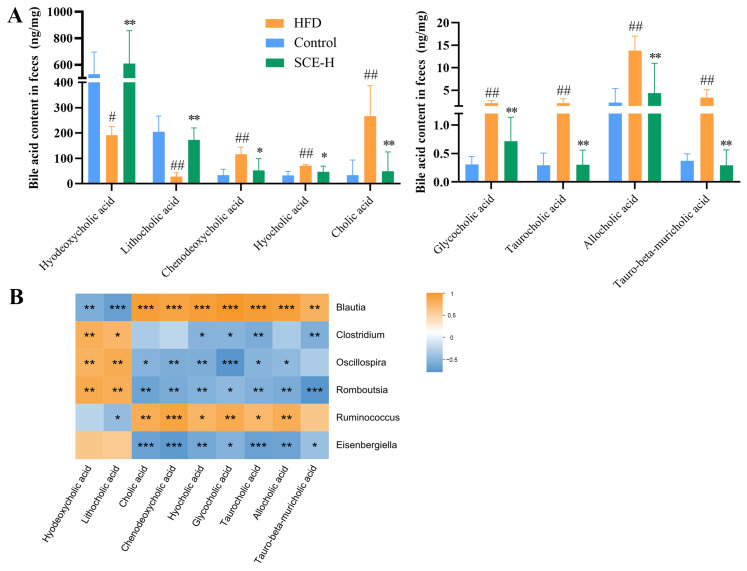
SCE changes the content of fecal BAs. (**A**) Levels of BAs in feces. (**B**) Spearman correlation analysis between gut microbiota and BAs. A positive correlation is displayed as orange, while a negative correlation is shown in blue. The significance is indicated as ^#^ *p* < 0.05, ^##^ *p* < 0.01 compared with the control group, * *p* < 0.05, ** *p* < 0.01, and *** *p* < 0.001, compared with the HFD group (In B, compared between gut microbiota and BAs).

We determined the contents of BAs in rat fecal samples using a targeted metabolomics approach based on UPLC-MS/MS. The results showed that the levels of T-β-MCA, TCA, GCA, ACA, CA, CDCA, and HCA in rat feces declined in the SCE group, and the contents of HDCA and LCA were elevated compared with those in the HFD group (*p* < 0.05, Figure 8A). The data analysis showed that Clostridium, Romboutsia, and Oscillospira were negatively associated with T-β-MCA, TCA, GCA, ACA, CA, CDCA, and HCA, as well as positively associated with HDCA and LCA. Meanwhile, Blautia and Ruminococcus were negatively associated with HDCA and LCA and positively correlated with T-β-MCA, TCA, GCA, ACA, CA, CDCA, and HCA (*p* < 0.05, Figure 8B). Changes in the BA composition further validated the untargeted metabolome results and confirmed that SCE modulated bile acid disorders in the HFD group of rats.

### 3.7. SCE Reduces Liver Lipid Deposition by Regulating Bile Acid Metabolism Pathway

**Figure 9:**
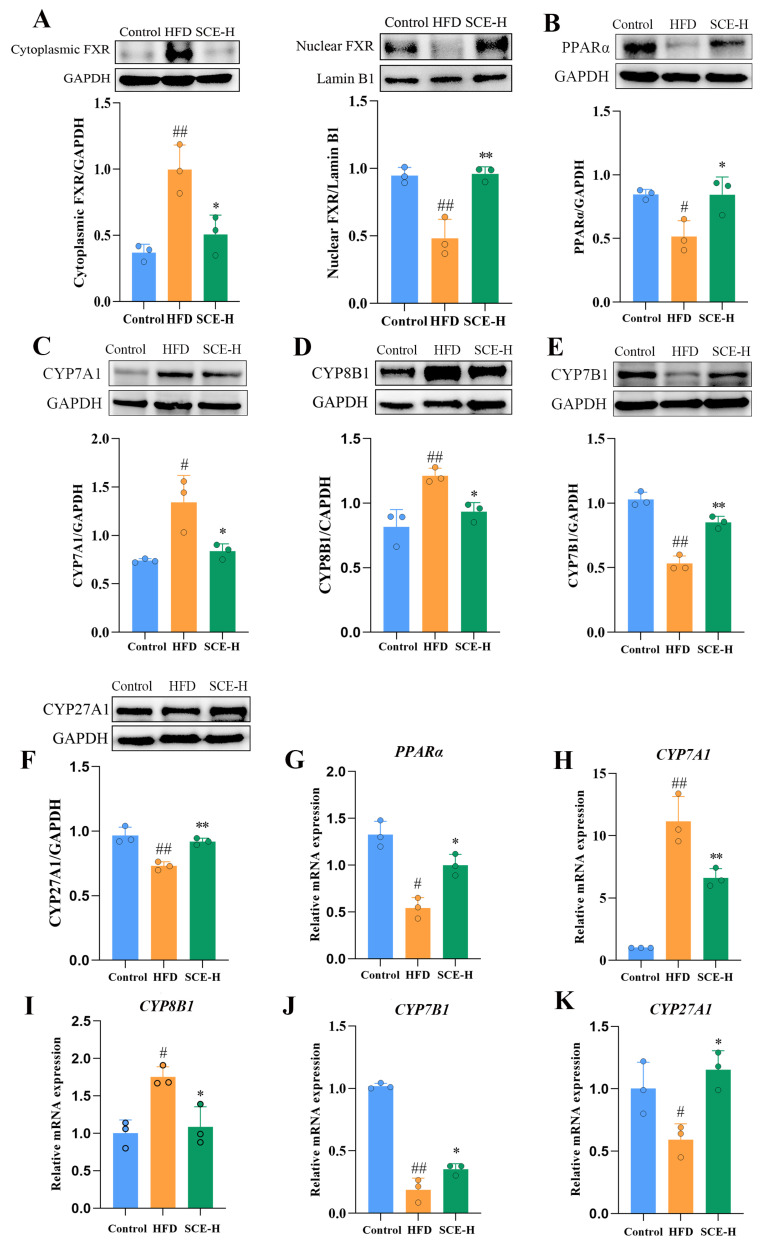
SCE activated the FXR/PPARα pathway and improved lipid metabolism. (**A**–**K**) The protein levels of FXR (**A**), PPARα (**B**), CYP7A1 (**C**), CYP8B1 (**D**), CYP7B1 (**E**), and CYP27A1 (**F**) determined via Western blot. (**G**–**K**) mRNA expressions of *PPARα* (**G**), *CYP7A1* (**H**), *CYP8B1* (**I**), *CYP7B1* (**J**), and *CYP27A1* (**K**) assessed via *RT-PCR*. Data represent the mean ± SD, ^#^ *p* < 0.05, and ^##^ *p* < 0.01, compared with the control group. * *p* < 0.05, and ** *p* < 0.01, compared with the HFD group (*n* = 3).

Based on the results of the metabolomics and 16S rDNA studies, we inferred that regulating bile acid metabolism potentially constitutes the mechanism underlying SCE therapeutic efficacy. The protein expression levels of FXR in the nucleus and cytoplasm of livers were first examined. As shown in Figure 9A, SCE treatment promoted the nuclear translocation of FXR. Our assay detected the abundance of mRNA and proteins that were correlated with two main pathways of bile acid biosynthesis, namely, CYP7A1, CYP7B1, CYP8B1, and CYP27A1. As illustrated in Figure 9C–F,H–K, SCE treatment significantly elevated liver bile acid receptor CYP7B1 and CYP27A1 levels in comparison with the HFD group and diminished the expression of CYP7A1 and CYP8B1, confirming the metabolomics findings (*p* < 0.05).

PPARα and FXR both function as transcriptional regulators of lipid metabolic genes, modulating hepatic lipid processing while coordinating bile acid production and transport [20]. These receptors are crucial regulators of both lipid metabolism and bile acid balance. Consequently, we examined the PPARα expression at both protein and mRNA levels. Figure 9B,G demonstrate that SCE-H administration markedly enhanced the PPARα protein and mRNA levels compared with the HFD group, suggesting PPARα activation (*p* < 0.05).

## 4. Discussion

A total of 39 compounds were recognized in the SCE samples, including 4-hydroxybenzoic acid, caffeic acid, resveratrol, oxyresveratrol, quercetin, rutin, gentianolic acid, and glycoside. Several studies have reported that many components can ameliorate lipid accumulation due to an HFD, such as caffeic acid [31], eriodictyol [32], quercetin [33], resveratrol [34], taxifolin [35], rutin [36], and cinchonine [37], all of which are contained in the alcoholic extract of *Smilax china* L. Gut microbiota restructuring is crucial for alleviating MAFLD. Notably, certain bioactive compounds in traditional Chinese medicine may influence the gut microbiota [38]. Mounting evidence suggests that products isolated or purified by foods may exert a biologically activity which is beneficial in a number of pathologic conditions [39]. In particular, traditional Chinese medicine offers different example of nutraceuticals which are effective in the prevention or treatment of chronic disorders affecting different organs. In this respect, a Chinese traditional medicine, i.e., Hericium erinaceus, has been described to affect gut microbiota thus potentially exerting a beneficial effect in MASLD [40]. Components of *Smilax china* L., such as arctigenin and catechin, can ameliorate obesity due to an HFD through modulating gut microbiota [41,42]. These components may be the therapeutic material that SCE exerts against MAFLD.

Accumulating evidence suggests that the gut microbiota help ameliorate MAFLD [43,44]. In vivo research has shown that *Blautia* increases liver inflammation and hepatic fibrosis in normal and MASH mice [45]. *Oscillospira* abundance has a favorable relationship with leanness [46]. *Romboutsia* is negatively correlated with obesity in bacteria upregulated by α-d-glucan [47]. In this study, SCE significantly decreased the *Blautia* level, while elevating the *Oscillospira* and *Romboutsia* levels. Spearman’s correlation analysis further showed that gut microbes (*Blautia*, *Clostridium*, *Oscillospira*, *Romboutsia*, *Ruminococcus*, and *Eisenbergiella*) were significantly associated with MAFLD-related biochemical markers. As shown by the TG, TC, MDA, SOD, GSH-Px, ALT, AST, LDL-C, and HDL-C, SCE can exert a liver-protective effect by regulating the gut microbiota to reduce liver lipid accumulation. The LPS was significantly correlated with all six genus level strains, whereas the inflammation markers IL-18, IL-6, and TNF-α were also differentially correlated with the described bacterial strains. SCE reduces intestinal permeability, thus decreasing LPS migration into the blood and liver, which leads to lower liver inflammation. Our findings demonstrate that SCE can improve MAFLD through modulating the gut microbiota to reduce inflammation.

Observational findings achieved during the last 20 years suggest that the gut microbiota likely participate in host metabolic processes [48]. Primary BAs are produced in hepatocytes and accumulated in the gallbladder, whereas secondary BAs are generated through intestinal bacterial metabolism. Additionally, BAs can influence microbial composition and function [49]. Our studies showed these metabolites were highly altered in MAFLD rats, but they significantly improved after SCE treatment, and secondary bile acid biosynthesis was the main metabolic pathway affected by SCE. Correlation analysis showed that differentially expressed flora and differentially expressed bile acids were correlated to varying degrees. Some researchers have shown that the proliferation of the bacterial genera capable of bile acid 7α-dehydroxylation, such as *Clostridium* and *Oscillospira*, enable the synthesis of the secondary bile acids HDCA and LCA in the gut [50,51]. Importantly, SCE significantly increased the abundance of *Clostridium* and *Oscillospira* in MAFLD rats, and Spearman’s correlation analysis showed that *Oscillospira* and *Clostridium* positively correlated with HDCA and LCA and negatively correlated with T-β-MCA. The results suggest that SCE may affect bile acid metabolism by regulating the gut microbiota.

Metabolic shifts characterized by elevated TC, TG, and primary BAs, along with reduced secondary BAs in the liver, contribute to hepatic lipid accumulation [52]. In this study, the HFD disrupted the bile acid biosynthesis pathway, causing disorder in bile acid metabolism, as evidenced by the downregulation of HDCA and LCA, as well as the upregulation of T-β-MCA, HCA, and others. Recent studies have revealed that HDCA can modulate the FXR/PPARα pathway and gut microbiota dysbiosis thereby alleviating MAFLD [53]. Primary BAs bind to glycine or taurine in the body to form bile salts. Some bile acids bind to taurine to form conjugated bile acids that are antagonists of FXRs [54]. The secondary bile acid HDCA serves as a natural FXR activator that downregulates hepatic bile acid synthesis. HDCA administration attenuates MAFLD progression in mice by stimulating PPARα-driven fatty acid oxidation pathways in hepatocytes [55]. LCA acts as an agonist of FXR to regulate bile acid synthesis through the FXR signaling pathway [56]. As an antagonist of FXR, increased T-β-MCA depresses liver FXR expression, thereby inhibiting FXR-SHP signaling and activating liver CYP7A1 expression [57]. The core features of the bile acid pool changes induced by SCE treatment are the elevated levels of natural FXR agonists (HDCA, LCA) and the reduced contents of the FXR antagonist (T-β-MCA). This shift creates a microenvironment conducive to nuclear receptor activation. BAs activate the nuclear receptor FXR, which subsequently modulates the homeostasis of lipids [58]. By activating FXR, MAFLD may be improved via reduced liver lipids, attenuated inflammation, and improved insulin sensitivity [59]. The activation of FXR feedback inhibits CYP7A1 expression, causing decreased bile acid synthesis and decreased cholestasis in hepatocytes [60]. As the rate-limiting enzyme in the alternative bile acid synthesis pathway, hepatic CYP7B1 exerts a pivotal function in MAFLD and diabetes progression, as well as their therapeutic modulation [59]. Activation of hepatic FXR can further inhibit the hepatic enzymes, CYP7A1 and CYP8B1, activity, and activation of CYP7B1 and CYP27A1 activity inhibits liver BA synthesis [61]. The expression level of liver FXR, CYP7B1, and CYP27A1 were markedly elevated in the SCE-treated HFD rats in comparison with the HFD group, and the expression of CYP7A1 and CYP8B1 were diminished by SCE treatment. PPARα regulates hepatic lipid metabolism, and activation may reduce hepatic lipid accumulation, inflammatory factor production, and oxidative stress [25]. Recent research has shown the inhibition of the hepatic CYP7A1-centered classical BA pathway via PPARα signaling in HFD mice following HDCA treatment [53]. Consistent with these results, we showed that SCE inhibited liver weight gain, ameliorated lipid accumulation, and modulated disturbances in bile acid metabolism through the activation of PPARα. In conclusion, the results demonstrate the value of SCE in ameliorating bile acid metabolism disorders in MAFLD through activation of PPARα. The potential mechanism by which SCE combats MAFLD involves modulation of the microbiota, thereby enhancing the production of bile acid receptor agonists (such as HDCA, LCA, and T-β-MCA), which subsequently activate the FXR/PPARα pathway to regulate bile acid metabolism.

## 5. Conclusions

**Figure 10:**
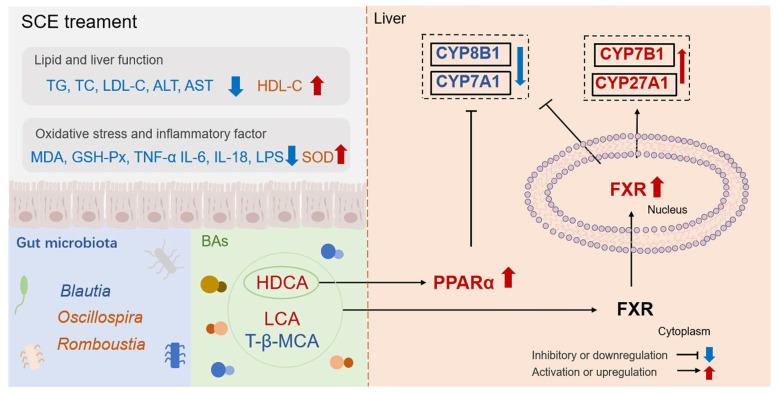
SCE alleviates MAFLD by regulating the gut microbiota imbalance and bile acid metabolism disorder and activating the FXR/PPARα pathway.

As shown in Figure 10, our study demonstrates for the first time that SCE can alleviate hepatic steatosis, inflammation, and oxidative stress induced by HFD in MAFLD rats, and the mechanism is related to the regulation of intestinal flora imbalance and bile acid metabolism disorder and to the activation of the FXR/PPARα pathway. This study reveals the positive impact of SCE on remodeling the gut microbiota and protecting against disorders of bile acid metabolism, providing valuable insights into the potential development and pragmatic application of SCE.

## Data Availability

The data that support the findings of this study are available on request from the corresponding author upon reasonable request.

## Supplementary Materials

The following supporting information can be downloaded at: https://www.mdpi.com/article/10.3390/metabo16010031/s1, Figure S1: Chemical profiles of SCE analyzed using UPLC-Q-TOF-MS. (A) Total ion chromatogram of SCE; (B–D) Extracted ion Chromatograms of identified compounds in positive mode. Table S1: Bile acids standard product information. Table S2: Primers used for RT-PCR analysis. Table S3: Data from UPLC-QTOF/MS/MS on the major SCE components in the positive mode. Table S4: Data from UPLC-QTOF/MS/MS on the major SCE components in the negative mode. Table S5: The differentially expressed metabolites. Table S6: The differentially expressed metabolites.

## Abbreviations

The following abbreviations are used in this manuscript:

ALT, alanine aminotransferase; AST, aspartate aminotransferase; BAs, bile acids; CYP7A1, cholesterol 7 alpha-hydroxylase; CYP27A1, mitochondrial sterol 27-hydroxylase; CYP7B1, 25-hydroxycholesterol 7-alpha-hydroxylase; CYP8B1, Cytochrome P450 8B1; FXR, farnesoid X receptor; GSH-Px, glutathione peroxidase; HDCA, hyodexycholic acid; HDL-C, high-density lipoprotein; HFD, high-fat diet; H&E, hematoxylin and eosin; LDA, linear discriminant analysis; LDL-C, low-density lipoprotein; LEfSe, linear discriminant analysis effect size; IL-6, interleukin-6; IL-18, interleukin-18; LPS, lipopolysaccharides; MAFLD, Metabolic-associated fatty liver disease; MDA, malondialdehyde; OPLS-DA, orthogonal partial least squares-discriminant analysis; PCA, principal component analysis; PPAR-α, peroxisome proliferator-activated receptor α; q-RT PCR, reverse transcription-quantitative polymerase chain reaction; SCE, *Smilax china* L. extract; SOD, superoxide dismutase; TC, total cholesterol; TG, triglycerides; TNF-α, tumor necrosis factor-α; UPLC-Q-TOF-MS, ultra-high-performance liquid chromatography with quadrupole time-of-flight mass spectrometry; VIP, variable importance in the projection.
