# Supplementary material for: Smilax china L. Extract Alleviates Metabolic-Associated Fatty Liver Disease by Regulating Gut Microbiota and Bile Acid Metabolism
Source: Metabolites. 2025 Dec 26;16(1):31. doi: 10.3390/metabo16010031 (PMC12844446; doi:10.3390/metabo16010031)

Table S1: Bile acids standard product information.

No.	Name	Abbreviation	CAS Number	Molecular Formula
1	Glycocholic Acid	GCA	475-31-0	$C_{26}H_{43}NO_6$
2	Glycochenodeoxycholic Acid	GCDCA	16564-43-5	$C_{26}H_{42}NO_5$
3	Taurocholic Acid	TCA	145-42-6	$C_{26}H_{44}NO_7S$
4	Taurochenodeoxycholic Acid	TCDCA	516-35-8	$C_{26}H_{45}NO_6S$
5	Glycoursodeoxycholic Acid	GUDCA	64480-66-6	$C_{26}H_{43}NO_5$
6	Allocholic Acid	ACA	2464-18-8	$C_{24}H_{40}O_5$
7	Ursodeoxycholic Acid	UDCA	128-13-2	$C_{24}H_{40}O_4$
8	Deoxycholic Acid	DCA	83-44-3	$C_{24}H_{40}O_4$
9	Tauroursodeoxycholic Acid	TUDCA	14605-22-2	$C_{26}H_{45}NO_6S$
10	Hyodeoxycholic Acid	HDCA	83-49-8	$C_{24}H_{40}O_4$
11	Apocholic Acid	apoCA	641-81-6	$C_{24}H_{38}O_4$
12	Glycodeoxycholic Acid	GDCA	16409-34-0	$C_{26}H_{42}NO_5$
13	Glycolithocholic Acid	GLCA	474-74-8	$C_{26}H_{43}NO_4$
14	α-Muricholic Acid	α-MCA	2393-58-0	$C_{24}H_{40}O_5$
15	β-Muricholic Acid	β-MCA	2393-59-1	$C_{24}H_{40}O_5$
16	7-Ketolithocholic Acid	7-KLCA	4651-67-6	$C_{24}H_{38}O_4$
17	Tauro-α-Muricholic Acid	T-α-MCA	25613-05-2	$C_{26}H_{44}NO_7S$
18	Tauro-β-Muricholic Acid	T-β-MCA	145022-92-0	$C_{26}H_{44}NO_7S$
19	ω-Muricholic Acid	ω-MCA	6830/3/1	$C_{24}H_{40}O_5$
20	Murideoxycholic Acid	MDCA	668-49-5	$C_{24}H_{40}O_4$
21	Taurohyodeoxycholic Acid	THDCA	38411-85-7	$C_{26}H_{44}NO_6S$
22	Taurohyocholic Acid	THCA	N/A	$C_{26}H_{46}NO_7S$
23	Taurolithocholic Acid	TLCA	6042-32-6	$C_{26}H_{44}NO_5S$
24	Taurodeoxycholic Acid	TDCA	1180-95-6	$C_{26}H_{44}NO_6S$
25	Lithocholic Acid	LCA	434-13-9	$C_{24}H_{40}O_3$
26	Cholic Acid	CA	81-25-4	$C_{24}H_{40}O_5$

27	Chenodeoxycholic Acid	CDCA	474-25-9	$C_{24}H_{40}O_4$
28	Hyocholic Acid	HCA	547-75-1	$C_{24}H_{40}O_5$
29	23-Norcholic Acid	NorCA	60696-62-0	$C_{23}H_{38}O_5$
30	Glycohyocholic Acid	GHCA	32747-08-3	$C_{26}H_{43}NO_6$
31	23-Nordeoxycholic Acid	NorDCA	53608-86-9	$C_{23}H_{38}O_4$
32	Isolithocholic Acid	isoLCA	1534-35-6	$C_{24}H_{40}O_3$
33	12-Ketolithocholic Acid	12-KLCA	5130-29-0	$C_{24}H_{38}O_4$
34	Dehydrolithocholic Acid	DHLCA	1553-56-6	$C_{24}H_{38}O_3$
35	Lithocholic Acid 3-Sulfate	LCA-3S	34669-57-3	$C_{24}H_{38}O_6S$
36	Chenodeoxycholic Acid 3β-Glucuronide	CDCA-3Gln	58814-71-4	$C_{30}H_{48}O_{10}$
37	3β-Ursodeoxycholic Acid	3β-UDCA	78919-26-3	$C_{24}H_{40}O_4$
38	3-Dehydrocholic Acid	3-DHCA	2304-89-4	$C_{24}H_{38}O_5$
39	Chenodeoxycholic Acid 24-Acyl-β-D-Glucuronide	CDCA-24A-βGlu	208038-27-1	$C_{30}H_{48}O_{10}$
40	12-Oxochenodeoxycholic Acid	12-KCDCA	2458/8/4	$C_{24}H_{38}O_5$
41	7,12-Diketolithocholic Acid	7,12-DKLCA	517-33-9	$C_{24}H_{36}O_5$
42	Dehydrocholic Acid	DHCA	81-23-2	$C_{24}H_{34}O_5$
43	Ursocholic Acid	UCA	2955-27-3	$C_{24}H_{40}O_5$
44	7-Ketodeoxycholic Acid	7-KDCA	911-40-0	$C_{24}H_{38}O_5$
45	Isodeoxycholic Acid	isoDCA	566-17-6	$C_{24}H_{40}O_4$
46	3β-Cholic Acid	βCA	3338-16-7	$C_{24}H_{40}O_5$
47	Tauro-ω-Muricholic Acid	T-ω-MCA	N/A	$C_{26}H_{44}NO_7S$
IS-1	Cholic Acid-D4	CA-D4	99102-69-9	$C_{24}H_{36}D_4O_5$
IS-2	Chenodeoxycholic Acid-D4	CDCA-D4	116380-66-6	$C_{24}D_4H_{36}O_4$

Table S2: Primers used for RT-PCR analysis.

Gene	Direction	Primer sequence (5′– 3′)
PPARα	Forward	ATTTGCCAAGGCTATCCCA
	Reverse	GCATCCCGTCTTTGTTCATC
CYP7A1	Forward	ACAATTTGACCAAGTCTTTCCG
	Reverse	GGTTCTTGTGCTTCAATCCCT
CYP8B1	Forward	ACCTTCAAGTACAATCGGTTCC
	Reverse	TTCATCTCACTGAGGGCAAAG
CYP7B1	Forward	AAACCACAGTCGCATGTTTCT
	Reverse	TGCAGCCTTATTCCGCTAA
CYP27A1	Forward	AGTGGAAGGAGCACCGAGAC
	Reverse	GCATCCGCTGATTCAAACTAT
GAPDH	Forward	TCTCTGCTCCTCCCTGTTC
	Reverse	ACACCGACCTTCACCATCT

Table S3: Data from UPLC-QTOF/MS/MS on the major SCE components in the positive mode.

	Identification	Chemical formula	RT (min)	Neutral mass	Calculated (m/z)	Observed (m/z)	Error (ppm)	MS/MS
1	4-Hydroxybenzoic acid	$C_7H_6O_3$	2.261	138.0317	139.0395	139.0388	-0.5	119.0480，91.0534
2	Caffeic acid*	$C_9H_8O_4$	2.072	180.0423	181.0501	181.0502	0.6	163.0403，145.0283，91.0534
3	Resveratrol*	$C_{14}H_{12}O_3$	7.381	228.0786	229.0865	229.0855	-4.4	151.0409，107.0484，91.0534
4	Oxyresveratrol	$C_{14}H_{12}O_4$	5.796	244.0736	245.0814	245.0805	-3.7	153.0165，121.0277，107.0484
5	trans-Piceatannol	$C_{14}H_{12}O_4$	6.052	244.2427	245.0814	245.0805	-3.7	151.0409，121.0277，107.0484
6	Naringenin	$C_{15}H_{12}O_5$	9.088	272.0685	273.0763	273.0745	-6.6	167.0124，153.0165，107.0484
7	Kaempferol*	$C_{15}H_{10}O_6$	6.761	286.0477	289.0712	287.0562	2.1	165.0109，153.0165，107.0484，91.0534
8	Dihydrokaempferol	$C_{15}H_{12}O_6$	6.794	286.0477	289.0712	289.0707	-1.7	271.0594，191.0363，153.0165，107.0484
9	Eriodictyol	$C_{15}H_{12}O_6$	7.449	286.0477	289.0712	289.0707	-1.7	271.0594，153.0191，107.0484
10	(-)-catechin	$C_{15}H_{14}O_6$	4.19	290.2681	291.0869	291.0852	-5.8	179.084，151.0409，107.0484
11	Quercetin	$C_{15}H_{10}O_7$	6.605	302.2357	303.0505	303.0485	-6.6	153.0165，107.0484，105.0338，103.0550
12	Taxifolin	$C_{15}H_{12}O_7$	5.708	304.2516	305.0611	305.0645	-5.2	153.0165，107.0484，121.0277，105.0338，103.0550
13	Gentianolic acid glycoside	$C_{14}H_{20}O_8$	1.606	316.3038	317.1236	317.1229	-2.2	299.0540，145.0283，77.0383
14	Methyl gentianolic acid glycoside	$C_{14}H_{18}O_9$	2.949	330.2873	331.1029	331.1034	1.5	313.0691，151.0384，123.0442
15	Chlorogenic acid*	$C_{16}H_{18}O_9$	1.451	354.3087	355.1029	355.1013	-4.5	337.0956，107.0484，77.0383
16	Methyl chlorogenate	$C_{17}H_{20}O_9$	1.708	368.3353	369.1186	369.1155	-8.4	163.0377，103.0530，91.0533
17	Quercitrin*	$C_{21}H_{20}O_{11}$	6.639	448.3769	449.1084	449.1074	-2.2	433.0941，153.0165
18	Cynaroside	$C_{21}H_{20}O_{11}$	6.518	448.3769	449.1084	449.1074	-2.2	433.0941，153.0165
19	Rutin*	$C_{27}H_{30}O_{16}$	5.02	610.5175	611.1612	611.1603	-1.5	151.0384，123.0442，107.0505
20	quercetin-3-O-β-D-allopyranoside	$C_{21}H_{20}O_{12}$	7.294	464.3763	465.1033	465.1068	7.5	151.0384，167.0706，107.0505，91.0534
21	Isorhamnetin	$C_{16}H_{12}O_7$	9.466	316.2623	317.0661	317.0646	-4.7	167.0706，107.0484，91.0534
22	Arctigenin	$C_{21}H_{24}O_6$	3.779	372.1573	373.1651	373.1643	-2.1	271.0594，203.0392.151.0384，105.0695
23	CinchonainIIa	$C_{39}H_{32}O_{15}$	4.331	740.1741	741.1819	741.1783	-4.9	191.0335，179.0348，153.0191，139.0388
24	CinchonainIIb	$C_{39}H_{32}O_{15}$	4.467	740.1741	741.1819	741.1783	-4.9	191.0363，179.0320，153.0191，139.0388，123.0442
25	Dihydrocaphen 3-O-β-D-glucoside	$C_{21}H_{22}O_{11}$	5.175	450.1162	451.124	451.1219	-4.7	153.0191，139.0388，123.0442，105.0338
26	Dihydrocaphen 5-O-β-D-glucoside	$C_{21}H_{20}O_{11}$	5.742	448.1006	449.1084	449.1074	-2.2	473.1025，153.0165，105.0338
27	Engeletin*	$C_{21}H_{22}O_{10}$	6.761	434.1213	435.1291	435.1279	-2.8	191.0335，153.0191.107.0484

28	Catechin-[8,7-e]-4β-(3,4-dihydroxyphenyl)-dihyro-2(3H)-pyranone	$C_{24}H_{20}O_9$	6.727	452.1107	453.1186	453.1191	1.1	153.0191，107.0505，91.0534
29	Isoengeletin	$C_{21}H_{22}O_{10}$	7.226	434.1213	435.1291	435.1279	-2.8	151.0384，139.0338，107.0484
30	Luteolin-3-O-α-L-rhamnopyranoside	$C_{21}H_{20}O_{10}$	7.347	432.1056	433.1135	433.1111	-5.5	151.0409，107.0484，91.0534
31	CinchonainIa	$C_{24}H_{20}O_9$	7.692	452.1107	453.1186	453.1148	-8.4	191.0335，151.0384，139.0388，123.0420，107.0484
32	CinchonainIb	$C_{24}H_{20}O_9$	7.847	452.1107	453.1186	453.1148	-8.4	167.0706，151.0409，123.0442，107.0484
33	Scirpusin A	$C_{28}H_{22}O_7$	8.434	470.1366	471.1385	471.1416	-5.9	215.0684，197.0595，185.0592，147.0661
34	Unknown	$C_{28}H_{22}O_7$	8.967	470.1366	471.1385	471.1416	-5.9	343.0770，177.0551，145.0283
35	Unknown	$C_{28}H_{22}O_7$	9.209	470.1366	471.1385	471.1416	-5.9	177.0551，145.0283，107.0484

* Compounds identified using reference compounds.

Table S4: Data from UPLC-QTOF/MS/MS on the major SCE components in the negative mode.

	Identification	Chemical formula	RT (min)	Neutral mass	Calculated (m/z)	Observed (m/z)	Error (ppm)	MS/MS
1	4-Hydroxybenzoic acid	$C_7H_6O_3$	1.656	138.0317	137.0239	137.0221	-13.1	119.0450, 91.0189
2	Caffeic acid*	$C_9H_8O_4$	2.017	180.0423	179.0344	179.0338	-3.4	163.0388, 145.0270, 91.0521
3	Resveratrol*	$C_{14}H_{12}O_3$	7.346	228.0786	227.0708	227.0711	1.3	185.0597, 164.0130
4	Oxyresveratrol	$C_{14}H_{12}O_4$	5.741	244.0736	243.0657	243.0624	-13.6	201.0150, 174.0303, 130.0395
5	trans-Piceatannol	$C_{14}H_{12}O_4$	6.051	244.0736	243.0657	243.0624	-13.6	174.0330, 159.0447, 130.0419
6	Naringenin	$C_{15}H_{12}O_5$	9.053	272.0685	271.0606	271.0585	-7.7	187.0373, 151.0035, 107.0099
7	Kaempferol*	$C_{15}H_{10}O_6$	6.517	286.0477	285.0399	285.0371	-9.8	259.0569, 243.0273, 200.9801
8	Dihydrokaempferol	$C_{15}H_{12}O_6$	6.638	286.0477	285.0399	285.0371	-9.8	259.0602, 243.0273, 180.0051
9	Eriodictyol	$C_{15}H_{12}O_6$	5.673	286.0477	285.0399	285.0371	-9.8	218.0501, 151.0010
10	(−)-catechin	$C_{15}H_{14}O_6$	2.243	290.0790	289.0712	289.0722	3.5	245.0811, 227.0711, 205.0504, 203.0700, 135.0445, 123.0441, 109.0276
11	Quercetin	$C_{15}H_{10}O_7$	8.223	302.0427	301.0348	301.0348	0.0	107.0142, 121.0262, 151.0010
12	Taxifolin	$C_{15}H_{12}O_7$	4.897	304.0583	303.0505	303.0492	-4.3	285.0406, 267.0545, 241.0488
13	Gentianolic acid glycoside	$C_{14}H_{20}O_8$	2.017	316.1158	315.1080	315.0875	1.9	153.0158, 180.0380, 219.0309
14	Methyl gentianolic acid glycoside	$C_{14}H_{18}O_9$	1.794	330.0951	329.0873	329.0845	-5.8	268.0293, 191.0551, 167.0349
15	Chlorogenic acid*	$C_{16}H_{18}O_9$	1.450	354.0951	353.0873	353.0861	-3.4	167.0322, 191.0551, 269.0394
16	Cryptochlorogenic acid	$C_{16}H_{18}O_9$	2.172	354.0951	353.0873	353.0861	-3.4	191.0551, 179.0388, 173.0456
17	Methyl chlorogenate	$C_{17}H_{20}O_9$	2.914	368.1107	367.1029	367.1022	-1.9	254.0549, 178.0214, 153.0158
18	Quercitrin*	$C_{21}H_{20}O_{11}$	6.570	448.1006	447.0927	447.0922	-1.1	301.0348, 300.0266, 271.0214, 243.0273, 151.0010
19	Cynaroside	$C_{21}H_{20}O_{11}$	6.725	448.1006	447.0927	447.0922	-1.1	258.0493, 208.0612
20	Rutin*	$C_{27}H_{30}O_{16}$	4.965	610.1534	609.1456	609.1434	-3.6	300.0266, 301.0348, 229.0555, 257.0437
21	quercetin-3-O-β-D-allopyranoside	$C_{21}H_{20}O_{12}$	5.397	464.0955	463.0877	463.0849	-6.0	151.0010, 167.0322, 107.0142
22	Isorhamnetin	$C_{16}H_{12}O_7$	9.431	316.0583	315.0505	315.0511	1.9	167.0349, 107.0120
23	Arctigenin	$C_{21}H_{24}O_6$	8.844	372.1573	371.1495	371.1349	1.9	271.0585, 203.0770, 151.0388
24	CinchonainIIa	$C_{39}H_{32}O_{15}$	4.277	740.1741	739.1663	739.1694	4.2	191.0551, 179.0310, 153.0158, 139.0158
25	CinchonainIIb	$C_{39}H_{32}O_{15}$	4.432	740.1741	739.1663	739.1694	4.2	191.0551, 179.0388, 153.0158, 139.0406
26	(E/Z)-Polydatin*	$C_{20}H_{22}O_8$	4.621	390.1315	389.1236	389.1243	1.8	227.0711, 242.0578
27	Dihydrocaphen 3-O-β-D-glucoside	$C_{21}H_{22}O_{11}$	5.174	450.1162	449.1084	449.1079	-1.1	287.0529, 269.0427, 153.0158
28	Dihydrocaphen 5-O-β-D-glucoside	$C_{21}H_{22}O_{11}$	5.673	450.1162	449.1084	449.1079	-1.1	287.0459, 269.0427, 259.0602, 153.0133

29	Dihydrocaphen 7-O-β-D-glucoside	$C_{21}H_{22}O_{12}$	6.517	450.1162	449.1084	449.1079	-1.1	303.0492, 285.0371, 178.9981, 151.0010, 125.0214
30	Kaempferol-7-O-β-D-glucoside	$C_{21}H_{20}O_{11}$	6.638	450.1162	449.1084	449.1079	-1.1	151.0010, 139.0358, 123.0441, 105.0321
31	Engeletin*	$C_{21}H_{22}O_{10}$	6.759	434.1213	433.1135	433.1133	-0.5	1911.0353, 153.0133, 107.0120
32	Isoengeletin	$C_{21}H_{22}O_{10}$	7.448	434.1213	433.1135	433.1133	-0.5	151.0388, 139.0382, 109.0276
33	Luteolin-3-O-α-L-rhamnopyranoside	$C_{21}H_{20}O_{10}$	7.191	434.1213	433.1135	433.1133	-0.5	151.0010, 107.0120, 137.0221
34	Catechin-[8,7-e]-4β-(3,4-dihydroxyphenyl)-dihyro-2(3H)-pyranone	$C_{24}H_{20}O_9$	5.086	452.1107	451.1029	451.1021	-1.8	151.0035, 107.0120, 91.0189
35	CinchonainIa	$C_{24}H_{20}O_9$	6.672	452.1107	451.1029	451.1021	-1.8	191.0325, 151.0388, 123.0441, 109.0276
36	CinchonainIb	$C_{24}H_{20}O_9$	7.656	452.1107	451.1029	451.1021	-1.8	151.0388, 123.0441, 109.0276
37	Scirpusin A	$C_{28}H_{22}O_7$	8.432	470.1366	469.1287	469.1286	-0.2	213.0533, 197.0599, 185.0597
38	Unknown	$C_{28}H_{22}O_7$	8.965	470.1366	469.1287	469.1286	-0.2	341.0652, 175.0394, 145.0270
39	Unknown	$C_{28}H_{22}O_7$	9.208	470.1366	469.1287	469.1286	-0.2	175.0394, 145.0270, 107.0381

* Compounds identified using reference compounds

Table S5: The differentially expressed metabolites.

	Metabolite	Formula	Retention time	Mode	M/Z	Trend (HFD/Control)	Trend (SCE-H/HFD)
1	Betagarin	$C_{18}H_{16}O_6$	4.6762	pos	346.1282	↓***	↑***
2	DHAP(10:0)	$C_{13}H_{25}O_7P$	3.4034	pos	325.139	↑***	↓**
3	N-Cyclohexylformamide	$C_7H_{13}NO$	1.0689	pos	145.1336	↑**	↓**
4	Homocitrulline	$C_7H_{15}N_3O_3$	1.5653	pos	231.1449	↑**	↓**
5	5-Hydroxy-L-tryptophan	$C_{11}H_{12}N_2O_3$	2.0059	pos	221.0919	↑***	↓**
6	Arecaidine	$C_7H_{11}NO_2$	2.0223	pos	283.1649	↓**	↑*
7	Gynocardin	$C_{12}H_{17}NO_8$	2.162	pos	345.1302	↑***	↓***
8	3-Hydroxydodec-9-enedioylcarnitine	$C_{19}H_{33}NO_7$	2.4466	pos	370.222	↑**	↓**
9	(Z)-Resveratrol 4'-glucoside	$C_{20}H_{22}O_8$	4.939	pos	408.1649	↑**	↓*
10	1,11-Undecanedicarboxylic acid	$C_{13}H_{24}O_4$	4.7916	pos	245.1745	↓***	↑*
11	Vanylglycol	$C_9H_{12}O_4$	3.8192	pos	167.0703	↑**	↓**
12	Tetrahydrocortisone	$C_{21}H_{32}O_5$	3.9343	pos	365.2317	↓***	↑**
13	DG(8:0/12:0/0:0)	$C_{23}H_{44}O_5$	5.6808	pos	442.3523	↑***	↓**
14	Deoxyspergualin	$C_{17}H_{37}N_7O_3$	5.7126	pos	370.2949	↑***	↓***
15	Aflatoxin B1 dialcohol	$C_{18}H_{18}O_6$	6.4168	pos	348.1437	↓***	↑**
16	3a,7b,12a-Trihydroxy-5a-Cholanoic acid	$C_{24}H_{40}O_5$	6.4725	pos	834.6084	↑***	↓***
17	Ricinoleic acid	$C_{18}H_{34}O_3$	6.4645	pos	316.2844	↑**	↓*
18	Palmitoyl-L-carnitine	$C_{23}H_{45}NO_4$	6.4567	pos	400.3417	↑***	↓**
19	Methyl (3b,11x)-3-Hydroxy-8-oxo-6-eremophilen-12-oate	$C_{16}H_{24}O_4$	5.8344	pos	245.1535	↑***	↓***
20	Palmitoylcarnitine	$C_{23}H_{45}NO_4$	5.7601	pos	400.3418	↑***	↓**
21	Corchoroside B	$C_{29}H_{42}O_8$	5.649	pos	536.3215	↑**	↓*
22	Cinnamaldehyde	C_9H_8O	5.5854	pos	133.0648	↓***	↑**
23	9(S)-HOTrE	$C_{18}H_{30}O_3$	5.5541	pos	333.1805	↓*	↑**
24	2-Hydroxyacetophenone	$C_8H_8O_2$	5.141	pos	137.0598	↑***	↓**
25	Apigenin 7,4'-dimethyl ether	$C_{17}H_{14}O_5$	5.0314	pos	299.0911	↑**	↓***
26	Norizalpinin	$C_{15}H_{10}O_5$	4.981	pos	271.0598	↑**	↓*
27	Delta-Valerolactam	C_5H_9NO	1.9344	pos	100.0761	↑***	↓***
28	Petasinine	$C_{13}H_{21}NO_3$	4.8717	pos	303.17	↑**	↓*
29	Luteolinidin	$C_{15}H_{10}O_5$	4.8717	pos	271.0598	↑***	↓***

30	Taurocholic Acid	$C_{26}H_{45}NO_7S$	5.6093	pos	480.2775	↑*	↓*
31	4-Hydroxyretinoic acid	$C_{20}H_{28}O_3$	4.3141	pos	349.2368	↓**	↑**
32	Vanillin Acetate	$C_{10}H_{10}O_4$	1.7726	pos	195.0652	↑**	↓**
33	Wistin	$C_{23}H_{24}O_{10}$	3.8346	pos	461.1437	↑***	↓*
34	Vanillin	$C_8H_8O_3$	3.8346	pos	185.0808	↑***	↓***
35	Polyethylene, oxidized	$C_{12}H_{20}O_5$	3.597	pos	209.1171	↑***	↓*
36	(-)-Jasmonic acid	$C_{12}H_{18}O_3$	3.3974	pos	211.1327	↑***	↓**
37	3-Hydroxy-DL-kynurenine	$C_{10}H_{12}N_2O_4$	2.9068	pos	225.0868	↓***	↑***
38	5-(2'-Carboxyethyl)-4,6-Dihydroxypicolinate	$C_9H_9NO_6$	1.99	pos	228.0501	↑***	↓***
39	Delphinidin	$C_{15}H_{11}O_7$	2.4881	pos	326.0371	↑***	↓**
40	Sarmentosin	$C_{11}H_{17}NO_7$	2.1691	pos	240.0864	↑*	↓*
41	4-Deacetylneosolaniol	$C_{17}H_{24}O_7$	2.0223	pos	341.1563	↑*	↓*
42	Propionylcarnitine	$C_{10}H_{19}NO_4$	2.6093	pos	218.1386	↑**	↓**
43	3'-Adenylic Acid	$C_{10}H_{14}N_5O_7P$	1.5733	pos	330.0594	↓***	↑***
44	5-Hydroxylysine	$C_6H_{14}N_2O_3$	1.524	pos	204.1342	↑***	↓*
45	Retinoyl b-glucuronide	$C_{26}H_{36}O_8$	1.1643	pos	261.1086	↑**	↓*
46	2',6'-Dihydroxy-4'-methoxyacetophenone	$C_9H_{10}O_4$	2.7323	pos	165.0546	↑***	↓***
47	Glutaric acid	$C_5H_8O_4$	0.8846	pos	174.0761	↑*	↓**
48	Methylimidazoleacetic acid	$C_6H_8N_2O_2$	0.6192	pos	123.0555	↑***	↓***
49	Citral propylene glycol acetal	$C_{13}H_{22}O_2$	5.1109	pos	175.1482	↓*	↑*
50	Cholic Acid	$C_{24}H_{40}O_5$	5.8418	pos	426.3212	↑***	↓*
51	Allocholic acid	$C_{24}H_{40}O_5$	6.385	pos	426.321	↑***	↓**
52	Dehydrocurdione	$C_{15}H_{22}O_2$	5.7443	pos	235.1691	↑***	↓***
53	Pyrrhoxanthinol	$C_{37}H_{46}O_5$	5.6729	pos	571.3471	↑***	↓**
54	4'-Hydroxy-5,7-dimethoxyflavan	$C_{17}H_{18}O_4$	5.6013	pos	269.1169	↓***	↑***
55	O-(17-Carboxyheptadecanoyl)carnitine	$C_{25}H_{47}NO_6$	5.5854	pos	496.3031	↑**	↓***
56	3,7-Dihydroxycholan-24-oic acid	$C_{24}H_{40}O_4$	5.8264	pos	785.5911	↓***	↑***
57	Dihydrobiopterin	$C_9H_{13}N_5O_3$	1.4505	pos	303.1184	↑**	↓**
58	Estrone	$C_{18}H_{22}O_2$	5.7522	pos	271.169	↑***	↓**
59	N-Acetylneuraminic Acid	$C_{11}H_{19}NO_9$	0.5954	pos	310.113	↓***	↑***
60	Methylimidazoleacetic acid	$C_6H_8N_2O_2$	0.6292	neg	139.0506	↑***	↓***
61	Dehydrocyanaropicrin	$C_{19}H_{20}O_6$	2.8183	neg	343.116	↑***	↓***
62	Sayanedin	$C_{17}H_{14}O_5$	5.4486	neg	297.0772	↓***	↑*

63	5-Hydroxyindoleacetic acid	$C_{10}H_9NO_3$	4.1248	neg	190.0506	↑***	↓*
64	Hyodeoxycholic Acid	$C_{24}H_{40}O_4$	5.7894	neg	783.5789	↓***	↑**
65	4-Hydroxystachydrine	$C_7H_{13}NO_3$	1.3001	neg	204.0875	↑***	↓***
66	Quinolinic Acid	$C_7H_5NO_4$	2.3792	neg	166.0141	↓**	↑**
67	Acetylhomoserine	$C_6H_{11}NO_4$	1.7799	neg	142.0503	↑*	↓**
68	2'-Hydroxyacetophenone	$C_8H_8O_2$	2.4006	neg	181.0502	↑**	↓***
69	Beta-D-Glucopyranuronic acid	$C_{13}H_{14}O_9$	2.6049	neg	359.0626	↑***	↓**
70	6-[4-(carboxymethyl)-2-hydroxyphenoxy]-3,4,5-trihydroxyoxane-2-carboxylic acid	$C_{14}H_{16}O_{10}$	2.7822	neg	389.073	↑***	↓***
71	2-Octenedioic acid	$C_8H_{12}O_4$	3.1012	neg	217.0717	↓***	↑**
72	Urothion	$C_{11}H_{11}N_5O_3S_2$	3.1114	neg	324.0234	↑**	↓*
73	N-Acetyl-L-phenylalanine	$C_{11}H_{13}NO_3$	3.4539	neg	252.088	↑*	↓**
74	Acetaminophen	$C_8H_9NO_2$	3.4759	neg	150.0555	↑***	↓**
75	Leucodopachrome	$C_9H_9NO_4$	3.4826	neg	176.0349	↓***	↑**
76	Leukoaminochrome	$C_8H_9NO_2$	3.6227	neg	196.0613	↑**	↓*
77	Vulgarole	$C_{12}H_{20}O_3$	3.7483	neg	257.1397	↑***	↓***
78	Gamma-Aminobutyric acid-betaxanthin	$C_{13}H_{16}N_2O_6$	3.812	neg	277.0832	↑***	↓***
79	Tryptophol	$C_{10}H_{11}NO$	3.9403	neg	206.0821	↑***	↓***
80	15-Deacetylneosolaniol	$C_{17}H_{24}O_7$	4.5425	neg	361.1298	↑***	↓**
81	11-Dehydro-thromboxane B2	$C_{20}H_{32}O_6$	5.3655	neg	349.2024	↓***	↑***
82	Taurine	$C_2H_7NO_3S$	1.2124	neg	124.0066	↑**	↓*
83	(Z)-4-Dodecenal	$C_{13}H_{24}O$	6.2523	neg	451.3797	↓**	↑**
84	Agavasaponin D	$C_{56}H_{90}O_{27}$	5.5382	neg	596.2769	↑***	↓***
85	Pinocembrin	$C_{15}H_{12}O_4$	5.5318	neg	255.0665	↑***	↓***
86	Undeca-4,6,8-trienedioylcarnitine	$C_{18}H_{27}NO_6$	5.5203	neg	352.1768	↑***	↓*
87	Genipin	$C_{11}H_{14}O_5$	4.3255	neg	225.0767	↑**	↑***
88	Sorgolactone	$C_{18}H_{20}O_5$	4.2416	neg	361.1298	↑**	↓***
89	P-Cresol glucuronide	$C_{13}H_{16}O_7$	4.0989	neg	283.0827	↑***	↓**
90	(x)-2-Heptanol glucoside	$C_{13}H_{26}O_6$	4.0316	neg	259.1554	↓***	↑*
91	Dihydroferulic acid 4-O-glucuronide	$C_{16}H_{20}O_{10}$	3.4187	neg	417.1042	↑***	↓***
92	Cynaroside A	$C_{21}H_{32}O_{10}$	3.2567	neg	443.1927	↑***	↓***
93	Methyl (3x,10R)-dihydroxy-11-dodecene-6,8-diynoate 10-glucoside	$C_{19}H_{26}O_9$	3.1933	neg	379.1385	↑**	↓*

94	Valtrate	$C_{10}H_{12}O_5$	4.2681	neg	233.0432	↑***	↓**
95	1-Salicylate glucuronide	$C_{13}H_{14}O_9$	2.91	neg	359.0627	↑***	↓***
96	1-METHYLXANTHINE	$C_6H_6N_4O_2$	2.4325	neg	165.0413	↑***	↓**
97	Biopterin	$C_9H_{11}N_5O_3$	1.8522	neg	236.079	↑***	↓***
98	1-Piperideine-2-carboxylic acid	$C_6H_9NO_2$	0.8877	neg	172.0611	↑*	↓**
99	N-Choloylglycine	$C_{26}H_{43}NO_6$	4.0845	neg	500.2769	↑***	↓***

Table S6: The differentially expressed metabolites.

NO.	Metabolites	Formula	M/Z	Trend (HFD/Control)	Trend (SCE-H/HFD)
1	Tetrahydrocortisone	$C_{21}H_{32}O_5$	365.2317	↓***	↑**
2	3a,7b,12a-Trihydroxy-5a-Cholanoic acid	$C_{24}H_{40}O_5$	834.6084	↓**	↑***
3	Palmitoyl-L-carnitine	$C_{23}H_{45}NO_4$	400.3417	↑***	↓*
4	Palmitoylcarnitine	$C_{23}H_{45}NO_4$	400.3418	↑***	↓*
5	9(S)-HOTrE	$C_{18}H_{30}O_3$	333.1805	↓**	↑***
6	Taurocholic Acid	$C_{26}H_{45}NO_7S$	480.2775	↑***	↓*
7	(-)-Jasmonic acid	$C_{12}H_{18}O_3$	211.1327	↑***	↓**
8	Glutaric acid	$C_5H_8O_4$	174.0761	↑***	↓*
9	Cholic Acid	$C_{24}H_{40}O_5$	426.3212	↑***	↓**
10	Allocholic acid	$C_{24}H_{40}O_5$	426.3210	↑***	↓*
11	3,7-Dihydroxycholan-24-oic acid	$C_{24}H_{40}O_4$	785.5911	↓***	↑***
12	Estrone	$C_{18}H_{22}O_2$	271.1690	↑***	↓**
13	Hyodeoxycholic Acid	$C_{24}H_{40}O_4$	783.5789	↓***	↑***
14	11-Dehydro-thromboxane B2	$C_{20}H_{32}O_6$	349.2024	↓***	↑***
15	Taurine	$C_2H_7NO_3S$	124.0066	↑***	↓*
16	N-Choloylglycine	$C_{26}H_{43}NO_6$	500.2769	↑***	↓*

Figure S1: Chemical profiles of SCE analyzed using UPLC-Q-TOF-MS. (A) Total ion chromatogram of SCE; (B-D) Extracted ion Chromatograms of identified compounds in positive mode.

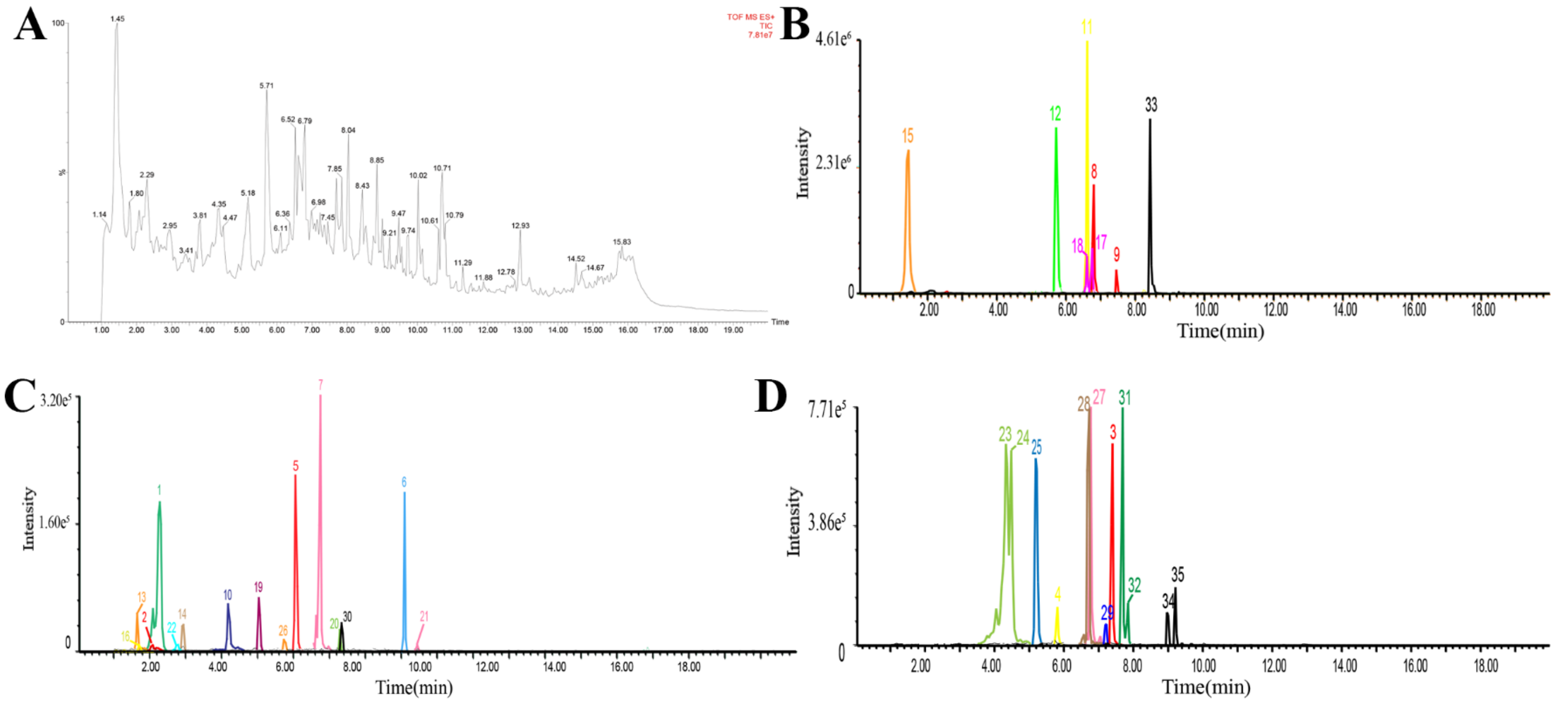

Supplement: Supplementary file 1 [file metabolites-16-00031-s001.zip › metabolites-4055969-Supplementary Materials.pdf]
